# Chloroplast characterizations and phylogenetic position of an endangered orchid, *Vanda coerulea* (Orchidaceae)

**DOI:** 10.1080/23802359.2019.1703600

**Published:** 2019-12-18

**Authors:** Qing-hua Zhang, Shu-zhao Zheng, Xiao-ting Wang, Wei-yao Zhu, Li-zhen Feng

**Affiliations:** Forestry College, Fujian Agriculture and Forestry University, Fuzhou, China

**Keywords:** *Vanda coerulea*, Orchidaceae, chloroplast genome, phylogenetic analysis

## Abstract

*Vanda coerulea* possess a high ornamental value and medical effect against glaucoma and cataract. The whole complete chloroplast (cp) genome of *V. coerulea* and the phylogenetic position based on the cp sequences remain unclear. Herein, we report the complete chloroplast genome of *V. coerulea*. The chloroplast genome was 149,376 bp in length, including a large single-copy (LSC) region of 86,100 bp, a small single-copy (SSC) region of 11,702 bp, and two inverted repeat (IRs) regions of 25,787 bp. A total of 129 genes were characterized, including 74 protein-coding genes, 36 tRNA genes, and 8 rRNA genes. The overall GC content was 36.6%, and GC percentages range from 27.9% to 43.2% throughout LSC, IRs, and SSC regions. Phylogenetic analysis based on 20 chloroplast genomes of Orchidaceae indicated that *V. coerulea* is closely related to *V. brunnea*. Our study provides a valuable resource for the identification and distinction of *Vanda* genus, and will lay a foundation for further research and conservation measures of *V. coerulea.*

*Vanda coerulea* is a perennial epiphyte belong to Orchidaceae family, it is mainly distributed in southern Yunnan, northeastern India, Myanmar and Thailand, and often grows on tree trunks in open forests or along rivers at an altitude of 60–100 m (Chen and Alexandra 2009). *Vanda coerulea* known as the Blue Vanda of Asia, it has a high ornamental value with sky-blue tessellated sepals and petals (Malabadi et al. 2004), and its flower juice is used as eye drops against glaucoma, cataract (Hossain 2011). Due to two threatening factors, over exploitation and habitat destruction, *V. coerulea* has been an endangered orchid in China (Wang and Xie 2004) and India (Pradhan 1985). To better understand of *V. coerulea*, we established the complete chloroplast genome of *V. coerulea* in this study. Our work will provide a valuable resource for the identification and distinction of *Vanda* genus, and will lay a foundation for further research and conservation measures of *V. coerulea.*

The samples of *V. coerulea* were collected from Menghai County, Xishuangbanna Dai Autonomous Prefecture, Yunnan Province, China (location: 21°56′51″N, 100°27′45″E). The specimens are kept in the Herbarium of Fujian Agriculture and Forestry University and can be acquired by the voucher specimen code FAFU08138.

The total genomic DNA was extracted from fresh leaves by modified CTAB method (Doyle and Doyle 1987), and sequenced by the BGISEQ-500 platform with PE150 pair-end library strategy (Mak et al. 2017). After removing adapters and low-quality reads, the clean reads were used to assemble the complete chloroplast genome by using GetOrganelle v1.5.2 (Jin et al. 2018), with the chloroplast genome of *V. brunnea* (no. MK442937) as the reference sequence. Then, the assembled chloroplast genome was annotated by using the Geneious R11.15 (Kearse et al. 2012). Finally, a complete chloroplast genome of *V. coerulea* with annotation information was obtained and submitted to GenBank with an accession number of MN711649.

The whole chloroplast genome of *V. coerulea* was 149,376 bp in length, including a large single-copy (LSC) region of 86,100 bp, a small single-copy (SSC) region of 11,702 bp, and two inverted repeat (IR) regions of 25,787 bp. A total of 129 genes were characterized, including 74 protein-coding genes, 36 tRNA genes, and 8 rRNA genes. The overall GC content was 36.6%, whereas the corresponding values of the LSC, SSC, and IR regions are 33.8%, 27.9%, and 43.2%, respectively.

To confirm the phylogenetic position of *V. coerulea*, 19 reported chloroplast genome sequences of Orchidaceae were downloaded from NCBI GenBank, including *V. brunnea* (MK442937), *Holcoglossum nyjiangense* (MK442930), *H. weixiense* (MK442936), *H. sinicum* (MK442933), *H. flavescens* (MK442925), *H. rupestre* (MK442932), *H. quasipinifolium* (MK442931), *H. lingulatum* (MK460222), *H. amesianum* (MK442924), *H. nagalandense* (MK442928), *H. wangii* (MK442935), *H. subulifolium* (MK442934), *Pendulorchis himalaica* (MK442927), *Neofinetia richardsiana* (KT726908), *N. falcata* (KT726909), *Pelatantheria scolopendrifolia* (KX871232), *Gastrochilus fiuscopunctatus* (KX871233), *Phalaenopsis equestris* (JF719062), and *Thrixspermum japonicum* (KX871234). All sequences were aligned with the HomBlock pipeline (Bi et al. 2018) and checked manually in Bioedit v5.0.9 (Hall 1999). Then, a maximum-likelihood (ML) tree was constructed with 1000 bootstrap replicates through the RAxML-HPC program on CIPRES Science Gateway (https://www.phylo.org). The phylogenetic analysis was shown in Figure 1, as excepted, the *V. coerulea* is most closely related to *V. brunnea*.

**Figure 1.**
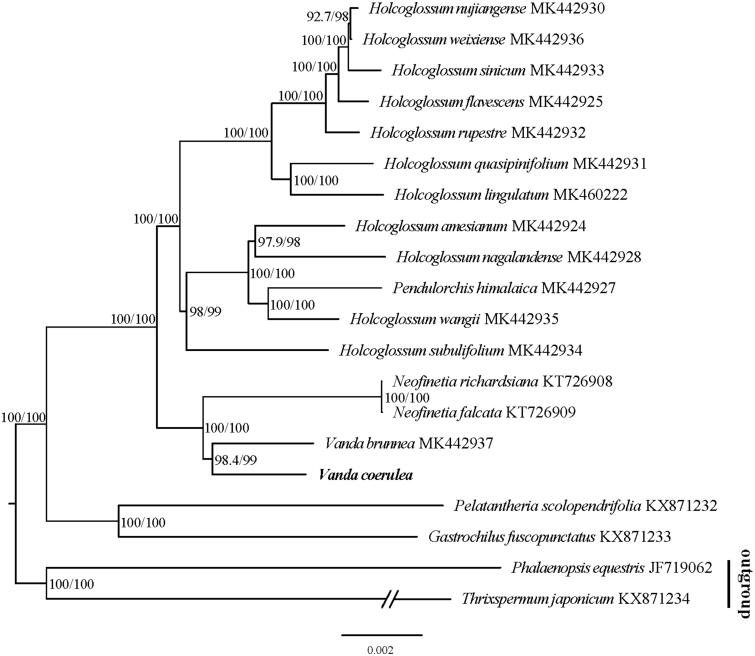
The maximum-likelihood (ML) phylogenetic tree of 20 selected Orchidaceae chloroplast sequences with 1000 bootstraps. All the sequences were downloaded from NCBI GenBank, the accession numbers were listed together with their species names, and the outgroup is presented at the bottom of the phylogenetic tree.
